# Human and viral membrane–associated E3 ubiquitin ligases MARCH1 and MIR2 recognize different features of CD86 to downregulate surface expression

**DOI:** 10.1016/j.jbc.2021.100900

**Published:** 2021-06-19

**Authors:** Raphael Trenker, Xinyu Wu, Julie V. Nguyen, Stephen Wilcox, Alan F. Rubin, Matthew E. Call, Melissa J. Call

**Affiliations:** 1 Structural Biology Division, The Walter and Eliza Hall Institute of Medical Research, Parkville, Victoria, Australia; 2 Department of Medical Biology, University of Melbourne, Parkville, Victoria, Australia; 3 Genomics Laboratory, The Walter and Eliza Hall Institute of Medical Research, Parkville, Victoria, Australia; 4 Bioinformatics Division, The Walter and Eliza Hall Institute of Medical Research, Parkville, Victoria, Australia

**Keywords:** membrane-associated E3 ubiquitin ligase, deep mutational scanning, protein–protein interactions, immune regulation, Kaposi's sarcoma virus, membrane protein, DMS, deep mutational scanning, DOX, doxycycline, HLA, human leukocyte antigen, ICAM-1, intercellular adhesion molecule 1, IRES, internal ribosome entry site, JM, juxtamembrane, KSHV, Kaposi's sarcoma herpesvirus, MARCH, membrane-associated RING-CH, MFI, mean fluorescence intensity, MHC, major histocompatibility complex, MIR, modulator of immune recognition, PolyVal TMD, PolyValine TM domain, TM, transmembrane, TMD, transmembrane domain, UMI, unique molecular identifier

## Abstract

Immune-stimulatory ligands, such as major histocompatibility complex molecules and the T-cell costimulatory ligand CD86, are central to productive immunity. Endogenous mammalian membrane-associated RING-CHs (MARCH) act on these and other targets to regulate antigen presentation and activation of adaptive immunity, whereas virus-encoded homologs target the same molecules to evade immune responses. Substrate specificity is encoded in or near the membrane-embedded domains of MARCHs and the proteins they regulate, but the exact sequences that distinguish substrates from nonsubstrates are poorly understood. Here, we examined the requirements for recognition of the costimulatory ligand CD86 by two different MARCH-family proteins, human MARCH1 and Kaposi's sarcoma herpesvirus modulator of immune recognition 2 (MIR2), using deep mutational scanning. We identified a highly specific recognition surface in the hydrophobic core of the CD86 transmembrane (TM) domain (TMD) that is required for recognition by MARCH1 and prominently features a proline at position 254. In contrast, MIR2 requires no specific sequences in the CD86 TMD but relies primarily on an aspartic acid at position 244 in the CD86 extracellular juxtamembrane region. Surprisingly, MIR2 recognized CD86 with a TMD composed entirely of valine, whereas many different single amino acid substitutions in the context of the native TM sequence conferred MIR2 resistance. These results show that the human and viral proteins evolved completely different recognition modes for the same substrate. That some TM sequences are incompatible with MIR2 activity, even when no specific recognition motif is required, suggests a more complicated mechanism of immune modulation *via* CD86 than was previously appreciated.

Cell-surface ligands play crucial roles in controlling adaptive immunity by modulating lymphocyte function both at initial activation and during downstream effector functions such as the clearance of infected or damaged cells. These crucial molecular cues are regulated in part by a family of membrane-associated RING-CH (MARCH) E3 ubiquitin ligases that mark cell-surface proteins for endocytosis and degradation by attaching ubiquitin to their cytoplasmic tails (1, 2). MARCH E3 ligases regulate the levels of major histocompatibility complex (MHC) class I and II proteins, the T cell costimulatory ligand CD86, the T cell coreceptor CD4, intercellular adhesion molecule 1 (ICAM-1), and some cytokine receptors, among other potential substrates, most of which are single-spanning membrane proteins (3, 4, 5, 6, 7, 8, 9, 10). MARCH proteins thereby contribute to control of cell–cell contact, antigen presentation, and key accessory signals that are integral to T cell activation. The potent immune-modulating potential of MARCH proteins is underscored by the existence of virus-encoded homologs (11, 12) that support viral immune evasion by downregulating some of the same target molecules in infected cells to block T cell responses. The best studied viral MARCHs are Kaposi's sarcoma herpesvirus (KSHV) proteins K3 and K5, also known as modulators of immune recognition (MIR)1 and MIR2, which downregulate MHC-I (MIR1/2), CD86 (MIR2 only), and ICAM-1 (MIR2 only) (11, 12, 13, 14).

Most MARCH proteins share a common architecture consisting of an N-terminal cytoplasmic RING-CH domain, two transmembrane (TM) domains connected by a short extracellular loop, and a C-terminal cytoplasmic tail of varying length (Fig. 1*A*). MARCH/MIR substrates are marked for downregulation through ubiquitin transfer to acceptor lysines in their cytoplasmic tails mediated by the cytoplasmic RING-CH domains of the MARCH proteins (6). However, the intermolecular interactions governing substrate specificity are thought to be encoded in and near the membrane-spanning portions of MARCHs and their substrates (4, 13, 14, 15, 16, 17, 18, 19, 20). Several studies have shown that susceptibility to MARCH-mediated downregulation can be transferred to nonsubstrate proteins by replacing their TMDs with sequences from substrate proteins, provided there are properly situated acceptor lysines available in the chimeric substrate cytoplasmic tails (4, 14, 15, 19, 20). Little is known about precisely what features of substrate TMDs identify them as MARCH/MIR targets. Only one study has reported dependence of substrate downregulation on a specific TM sequence (17), showing that human MARCH8 requires an aromatic-rich stretch of five amino acids at the cytosolic end of the human leukocyte antigen (HLA)-DRβ TMD for recognition of DRαβ heterodimers.

**Figure 1:**
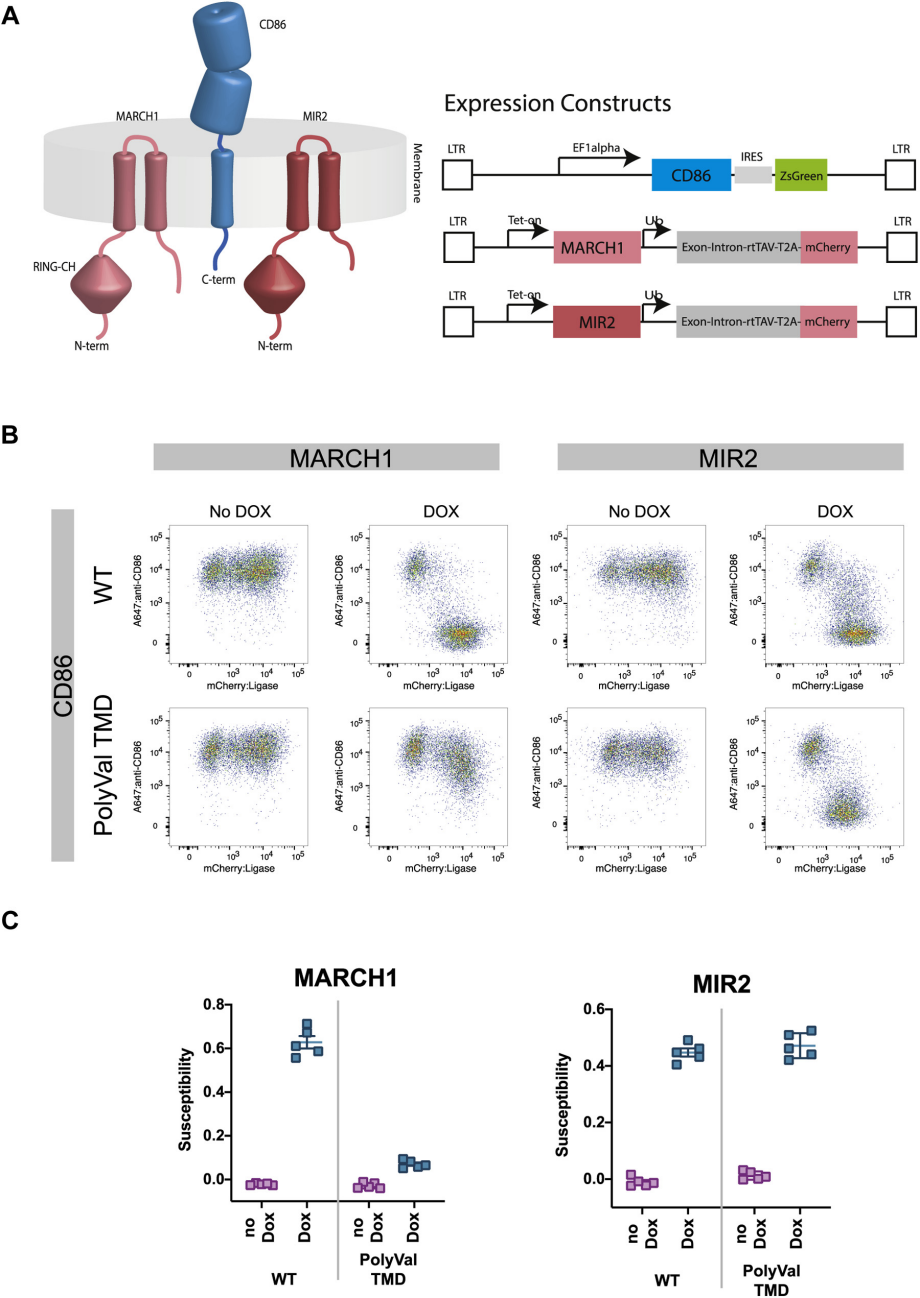
**MIR2 can regulate CD86 bearing a nondescript transmembrane domain, while MARCH1 cannot.***A*, *left*, cartoon showing architecture of CD86, MARCH1, and MIR2. *Right*, schematic of lentiviral expression vectors for constitutively expressed CD86- and DOX-inducible MARCH1 and MIR2. *B*, the ability of MARCH1 and MIR2 to downregulate WT CD86 was compared with their ability to downregulated CD86 carrying a polyvaline TMD. *C,* susceptibility scores calculated from five biological replicates (each done in technical triplicates) of the plots shown in *B*. MARCH1 is reliant on the TMD of CD86 for activity, whereas MIR2 is not. DOX, doxycycline; MARCH1, membrane-associated RING-CH; MIR2, modulator of immune recognition.

Here, we examine the question of TM sequence specificity in MARCH/MIR-mediated downregulation of human CD86. Like MHC-I and ICAM-1, CD86 is ubiquitinated, internalized, and lysosomally degraded in dendritic cells by both human MARCH and viral MIR proteins (15, 16). These share little sequence homology, raising the question of whether they use common or distinct recognition sequences to achieve the same functional outcome. Human MARCH1 and KSHV MIR2 are both active against human CD86, yet they share only 18% amino acid identity globally and only 17% in the TM–loop–TM regions believed to encode substrate selectivity, suggesting that they may use distinct features to recognize CD86. We interrogated the requirements for MARCH1 and MIR2 downregulation of CD86 under identical experimental conditions using both CD86 proteins with engineered polyvaline TM domains (PolyVal TMDs) and a deep mutational scanning (DMS) analysis (21, 22) of CD86 mutants in which every possible single amino acid substitution was included for the entire single TM and short juxtamembrane (JM) domains. Our results show that MARCH1 very specifically requires a proline at position 254 in the CD86 TMD, whereas MIR2 requires an aspartic acid in the extracellular JM domain, as previously reported in a study using a chimeric model substrate (18). Intriguingly, MIR2 requires no specific sequences in the CD86 TMD, because it effectively downregulated CD86 with a PolyVal TMD, yet single amino acid substitutions at many TM positions could impair susceptibility to MIR2 but not MARCH1. These results demonstrate that mammalian and viral MARCH-family proteins have evolved completely different recognition modes for the same crucial immune-stimulatory molecule.

## Results

### The human CD86 TMD is required for recognition by MARCH1 but not MIR2

Previous studies indicated that the recognition and downregulation of CD86 involved contributions from the TM and cytosolic domains for mouse MARCH1 (15) or the TM and extracellular JM regions for KSHV MIR2 (18). These studies used two different model chimeric substrates rather than full-length CD86, and the determinants of human MARCH1 specificity for human CD86 have never been systematically interrogated. We therefore tested dependence on the human CD86 TMD sequence for recognition by human MARCH1 and KSHV MIR2 in a quantitative cellular assay where they could be tested in parallel under identical experimental conditions (Fig. 1 and Fig. S1). We chose to assess MARCH1 and MIR2 functions in HeLa cells as they are naturally deficient in CD86 and amenable to retroviral transduction, allowing rapid generation of stable cell lines expressing WT CD86 and various mutants. Unmodified MARCH1 and MIR2 coding sequences were retrovirally introduced into HeLa cells under a tet-on element that provides doxycycline (DOX)-inducible expression (Fig. 1*A*). When these HeLa cells also stably express human CD86, DOX treatment causes removal of CD86 from the cell surface. This downregulation is measured using flow cytometry and converted into a susceptibility score that is proportional to the fraction of CD86 lost from the cell surface after MARCH induction (Fig. S1).

Two HeLa cell lines were generated that stably expressed either WT human CD86 or a variant in which all 19 amino acids in the TMD (between W248 and W268) were simultaneously replaced with valine (PolyVal TMD) (Fig. 1, *B* and *C*). Both CD86 proteins were expressed at similarly high levels on the HeLa cell surface. These cells were then transduced with DOX-inducible MARCH1 or MIR2 and analyzed for CD86 susceptibility. Both MARCH1 and MIR2 exhibited strong activity against WT CD86. CD86 PolyVal TMD was no longer effectively downregulated by MARCH1, experiencing only a small (but reproducible) reduction in cell-surface level after DOX treatment, but it retained a level of susceptibility to MIR2 that was indistinguishable from that of the WT CD86 protein. The dependence of MARCH1 on specific sequence elements in the CD86 TMD is therefore near complete, whereas MIR2 requires no particular CD86 TM sequences at all, with the possible exception that one of the four valines in the WT sequence is crucial. These starkly contrasting results demonstrate that MARCH1 and MIR2 use distinct recognition modes to downregulate CD86.

### Development and cell-surface expression profiling of a CD86 TM DMS library

**Figure 2:**
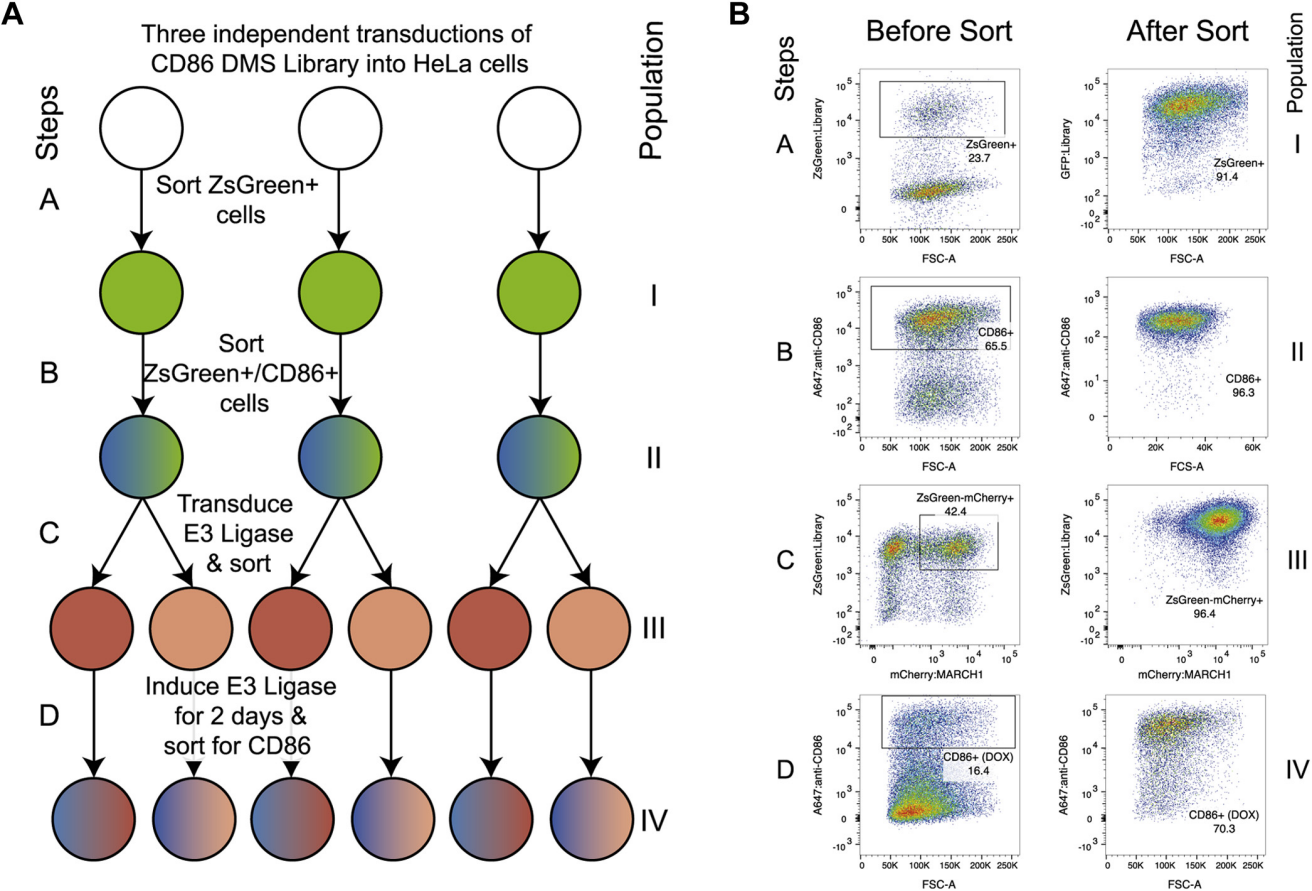
**Outline of the selection procedure of the deep mutational scan of the TM domain of CD86.***A*, experimental replicates and selection steps**.** HeLa cells were first transduced with the CD86 library and sorted on ZsGreen+ cells (Step A). ZsGreen+ cells were expanded on stained with Alexa-Fluor 647–labeled anti-CD86 to select CD86 variants that reach the cell surface (Step B). CD86+ cells were transduced with either MARCH1 and MIR2 and sorted on mCherry expression (Step C). Finally, doxycycline was added for 2 days, and cells that retained CD86 expression were enriched to select for variants that protect CD86 from MARCH1- or MIR2-mediated downregulation (Step D). *B*, representative flow cytometry plots of the selection procedure in panel *A* with steps and populations marked similarly as in panel *A*. MARCH1, membrane-associated RING-CH; MIR2, modulator of immune recognition; TM, transmembrane.

**Figure 3:**
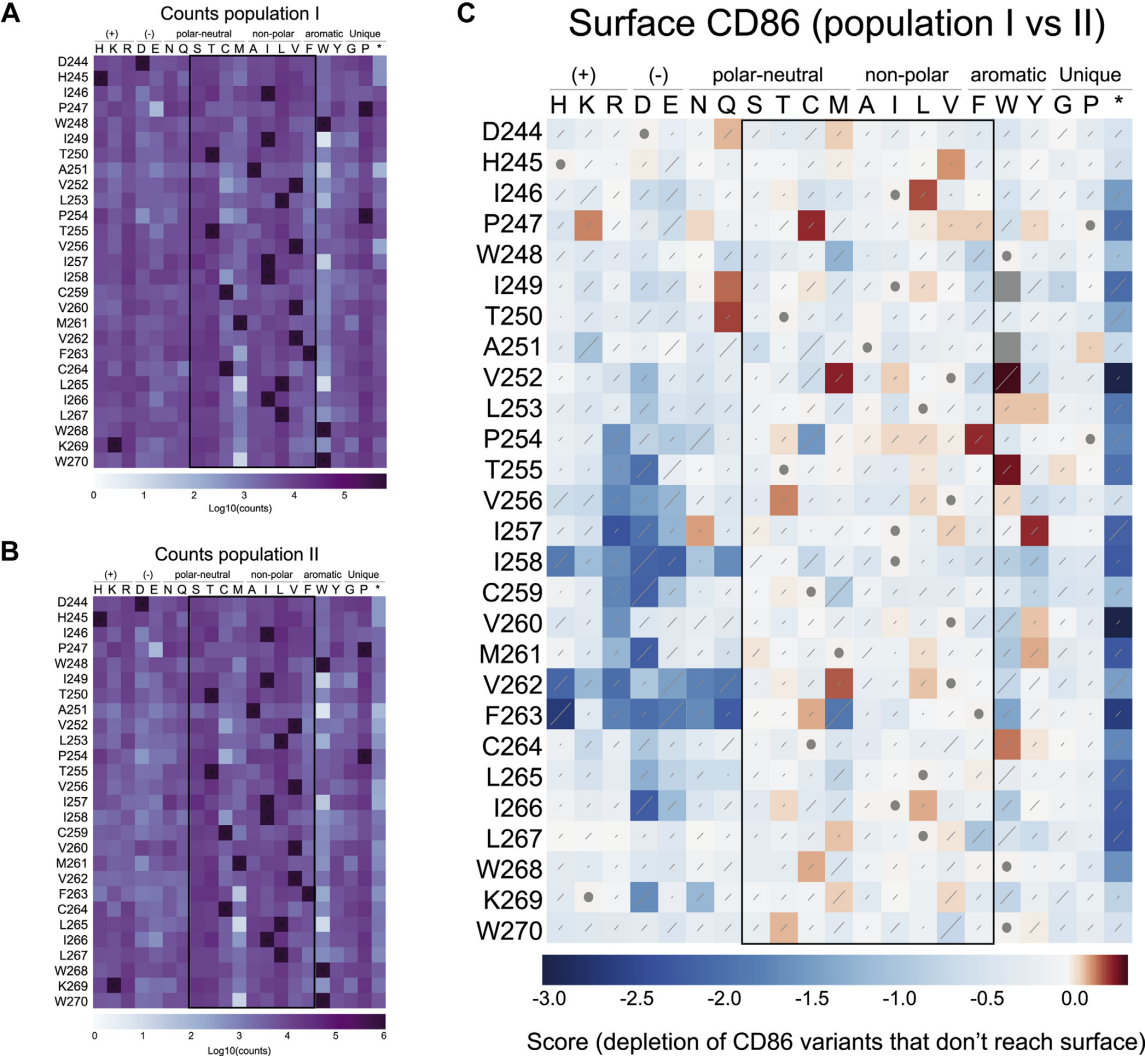
**Selection of CD86 TM variants that reach the cell surface.** Diversity plots show absolute counts of each variant before (*A*) and after (*B*) selection of cells based on surface levels of CD86, with populations referring to the workflow presented for Figure 2. The WT sequence is indicated by *squares with dots*. To generate the sequence-function heat map, each variant was given a log ratio score equal to log(variant counts/WT counts). Log ratio scores in the input population were subtracted from the log ratio scores of the selected population to yield the values presented for Figure 4. *C*, sequence function map of CD86 surface levels. Comparison of CD86 variant counts in the total ZsGreen+ population *versus* the ZsGreen+/CD86+ population details CD86 variants that are impaired in surface expression (*dark blue*). TM, transmembrane.

**Figure 4:**
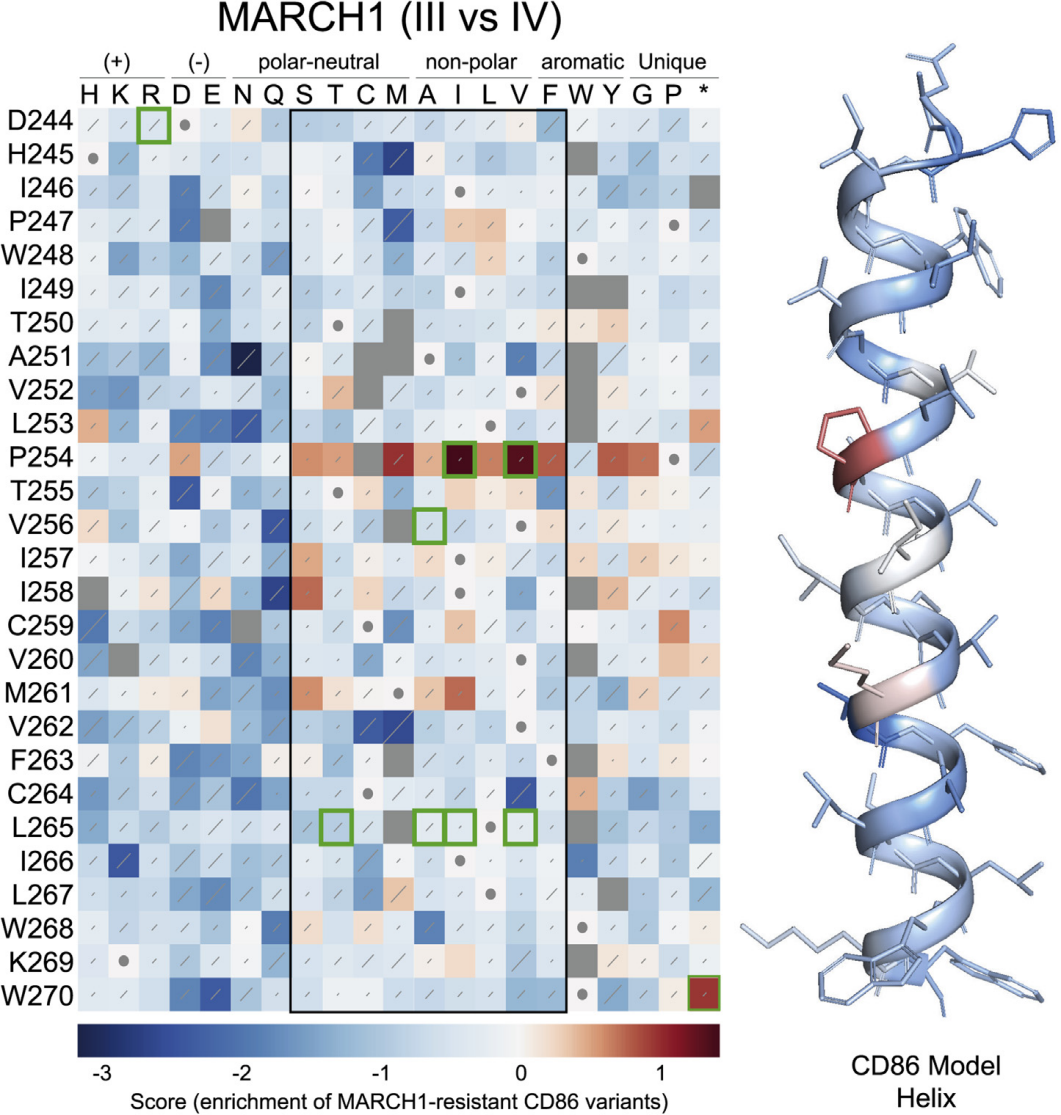
**Sequence-function map elucidating which CD86 mutants escape MARCH1 downregulation by comparing the frequency of population III and IV illustrated for**Figure 2**, *A* and *B*.** The WT sequence at each position is indicated by a *white square* with a *gray dot*. *Red squares* indicate variants that are protected from MARCH1-mediated downregulation, whereas *white* to *blue squares* indicate variants that are downregulated equally well or better than WT CD86. *Gray squares* indicate variants that are lost from the library during the selection process. *Diagonal lines* within each square represent standard errors, with *longer lines* indicating noisier data. A colourized CD86 model is shown where the shade of each residue is the average hue of the *squares* in the *black-boxed region* of the sequence-function map. Residues within the *black box* are easily accommodated in transmembrane domains. P254, I257, and M261 fall on one face, and mutations especially at P254 protect from MARCH1-mediated downregulation. Mutations selected for follow-up studies are boxed in *green*, and results are shown in Figure 6. MARCH1, membrane-associated RING-CH 1.

**Figure 5:**
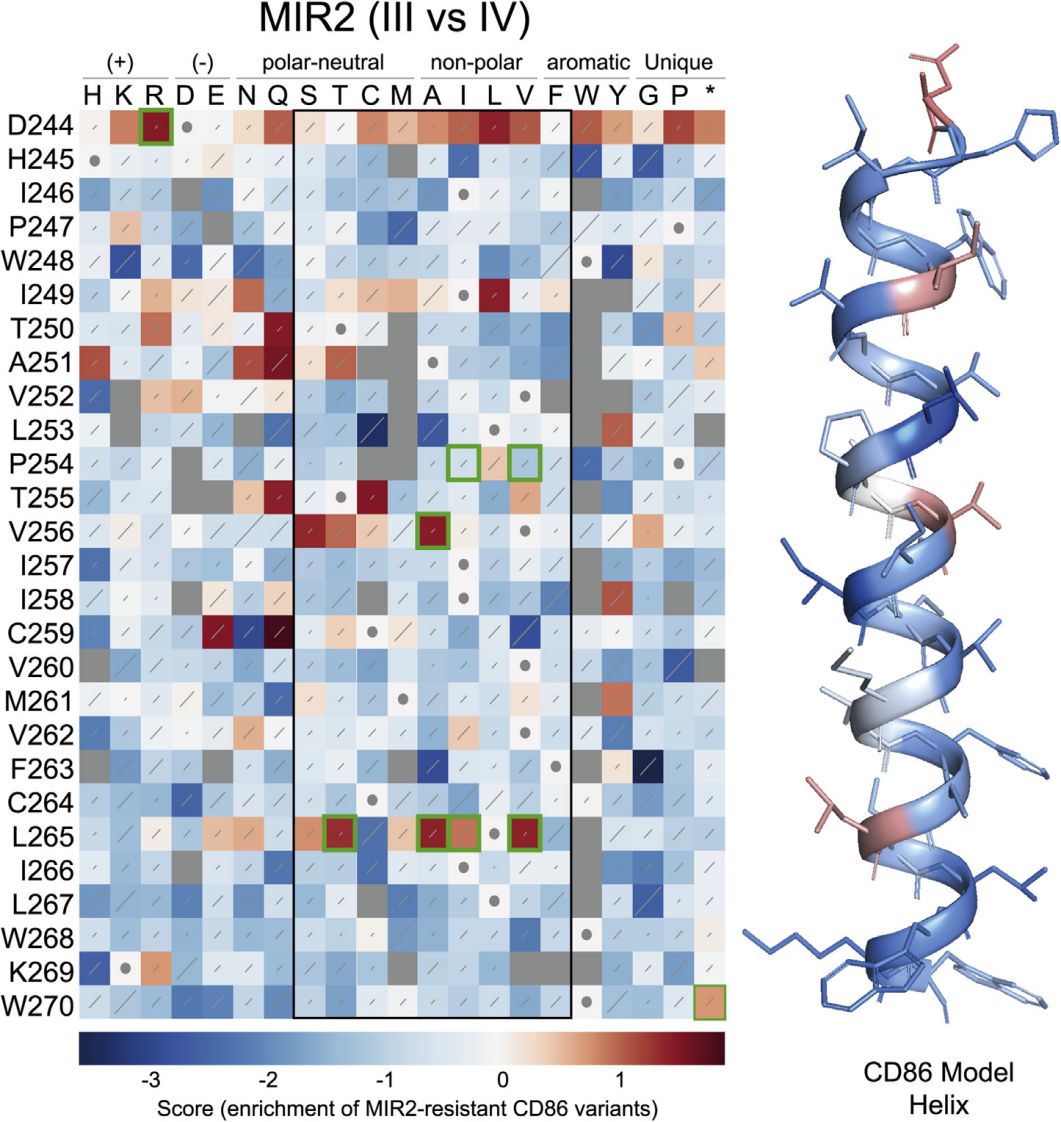
**Sequence-function map elucidating which CD86 mutants escape MIR2 downregulation by comparing the frequency of population III and IV as illustrated in**Figure 2**, *A* and *B*.** The WT sequence at each position is indicated by a *white square* with a *gray dot*. *Red squares* indicate variants that are protected from MARCH1-mediated downregulation, whereas *white* to *blue squares* indicate variants that are downregulated equally well or better than WT CD86. *Gray squares* indicate variants that are lost from the library during the selection process. *Diagonal lines within each square* represent standard errors, with *longer lines* indicating noisier data. A colourized CD86 model is shown where the shade of each residue is the average hue of the squares in the *black-boxed region* of the sequence-function map. Residues throughout the TM domain of CD86 protect from MIR2-mediated downregulation but are not clustered on any one face of the helix. Mutations selected for follow-up studies are boxed in *green*, and results are shown in Figure 6. MIR2, modulator of immune recognition 2; TM, transmembrane.

To interrogate the specific sequence requirements for recognition of the CD86 TMD, we devised a DMS approach that would yield the relative susceptibilities of all possible single amino acid substitutions in a 27-amino-acid stretch encompassing the 19 TM residues that had been replaced with valine in the aforementioned experiment, with an additional five extracellular and three intracellular JM positions to capture the effects of membrane-proximal mutations. A PCR-based strategy similar to one we previously reported (23) was employed to randomize one codon at a time using degenerate primers, yielding 1728 unique DNA sequences that encoded 567 protein variants, including early terminations and WT amino acid sequences. The combined variant library was retrovirally transduced into HeLa cells in triplicate and at low multiplicity of infection, with ZsGreen under an internal ribosome entry site (IRES) marking transduced cells. The resulting populations were sorted for ZsGreen expression (step A in Fig. 2). The ZsGreen^+^ cells (population I in Fig. 2) were expanded and further sorted for surface CD86 expression (step B). Total mRNA was prepared from samples of CD86^+^ cells (population II in Fig. 2) as well as total ZsGreen^+^ cells (population I), and the randomized region of CD86 was Illumina sequenced to measure variant frequencies before and after the CD86 sort. These are shown in diversity maps in Figure 3, *A* and *B* that represent variant counts as shades of purple in a matrix format for the entire sequence space covered in the screen. Each square represents the sum of counts for all synonymous codons encoding a given amino acid at that position. While most variants are well represented, some mutations show low counts both before and after selection and are therefore under-represented in the library. In particular, mutations to tryptophan and methionine can only be encoded by a single codon and are thus present at much lower frequencies than mutations to serine and arginine, which can both be encoded by six synonymous codons. Other examples are likely to be random variations arising during library preparation, such as P247E. Care should be taken when interpreting frequency changes in variants with low counts both before and after selection, as division by numbers approaching 0 can lead to large apparent differences. However, rather than cutoff data below a threshold number of counts, which can lead to meaningful changes being discarded when low frequency variants are enriched in subsequent steps, we have elected to display all data and assist the reader in evaluating sequence-function maps by including diversity maps calculated from an aggregation of the counts in all replicates before and after selection either in the main figure (Fig. 3) or in the supplement (Figs. 4 and 5).

Diversity data from preselection and postselection steps can be converted to a sequence-function heat map in Enrich2 (22) (Fig. 3*C*). Variants are scored based on the log of the variant counts divided by WT counts. These log ratios for the input population are subtracted from the log ratios for the selected population. Because WT counts are used in the ratio to normalize all variants, other factors such as loss of diversity and uneven variant representation are controlled for. Variants that are enriched or depleted at the same rate as WT have a score of 0 (set to *white* in the color scale). A variant that is enriched 10-fold relative to WT counts will have a score of 1 (*red*). A 10-fold depletion relative to WT conversely results in a score of −1 (*blue*). The resulting sequence-function heat map shows the effects of all TM/JM mutations on surface CD86 expression in cells that do not express MARCH1 or MIR2 (Fig. 3*C*).

This heat map reveals depletion of the types of variants one would expect when selecting for surface expression of a type I single-spanning membrane protein. For example, early termination mutants (*far right column*) are expected to be depleted because these remove or truncate the TMD, and most were strongly depleted (*dark blue squares*) compared with the WT sequence (*white squares* with *gray dots*) in population II. At some positions, early stop codons were only mildly depleted, for example, Asp244, His 245, Ala251, and Val256. These corresponded to variants that already had low starting frequencies (Fig. 3*A*) and therefore provide less reliable data. Also depleted were strong polar substitutions in the core hydrophobic region of the TMD (approximately A251–I266), since these substitutions carry large energetic penalties for residing in the nonpolar membrane interior (24). Most weakly polar and nonpolar substitutions (*boxed region* of the heat map) were well tolerated (*white squares*), as were glycine and proline substitutions, and a few scattered substitutions resulted in small increases in surface levels (*red squares*; note that the color scale is heavily weighted toward depletion). This analysis provides an important control to account for CD86 TM/JM substitutions that had particularly strong effects on surface display in the absence of MARCH/MIR overexpression and generated a starting population containing only variants that are surface expressed for our screens against MARCH1 and MIR2.

### MARCH1-mediated CD86 downregulation requires a proline at position 254 in the CD86 TMD

A pool of cells taken from this sorted CD86-positive population (Fig. 2*B*; step B, population II) was transduced with the DOX-inducible MARCH1 viral vector and sorted for its mCherry marker (step C in Fig. 2) to generate population III, which was essentially pure CD86^+^ MARCH1^+^ cells but did not yet express the MARCH1 protein. These cells were treated with DOX for 2 days in culture (step D in Fig. 2) to induce MARCH1 expression and then sorted a final time to isolate cells that remained CD86 positive (population IV); these cells harbored CD86 variants that had been rendered resistant to MARCH1-mediated downregulation by a single amino acid substitution somewhere in the TM/JM region. The randomized regions of the CD86 genes in these cells were sequenced as aforementioned to identify variants and measure their frequencies for comparison with those in the parent surface-CD86^+^ population III. The sequence-function heat map prepared from this analysis using Enrich2 (22) is shown in Figure 4 (diversity maps used to generate this figure are shown in Fig. S2). Although WT CD86 (*white squares with gray dots*) was strongly downregulated and therefore depleted from population IV compared with population III, enrichment scores have been normalized such that only variants that were enriched relative to WT appear in *shades of red*. These represent substitutions that inhibited MARCH1-mediated downregulation.

The clear enrichment of a premature stop codon at W270 verifies the ability of our screen to detect MARCH1-resistant CD86 variants. This protein contains the full TMD plus a tryptophan and lysine at the TM-to-cytosol transition, features that stabilize single-spanning proteins in the membrane, but it has lost the cytoplasmic tail containing most of the lysines that are the sites of ubiquitination (15, 25). Strikingly, most substitutions at P254 in the TMD also showed strong enrichment, indicating that they too were resistant to MARCH1 activity. Other substitutions that conferred some level of resistance were mostly concentrated in the core hydrophobic region, and the strongest effects occurred with helical periodicity from P254 at the +3, +4, and +7 positions (I257, I258, and M261, respectively). These data identify a clear face of the CD86 TM helix, with P254 as its most distinctive feature, which defines the recognition site for MARCH1.

### MIR2-mediated CD86 downregulation requires aspartic acid at position 244 in the CD86 extracellular JM region and is also sensitive to substitutions in the TMD

Our CD86 PolyVal TMD experiment (Fig. 1) indicated that there were no specific features in the TMD (other than general hydrophobicity) that were required for MIR2-mediated downregulation, yet previous studies had suggested a role for the TMD in addition to JM and cytosolic sequences (15, 18). We therefore performed the CD86 DMS screen for MIR2 in parallel with MARCH1 to get a more detailed view of its sequence dependence (Fig. 5; presort and postsort diversity maps in Fig. S3). The premature stop codon at W270 was again enriched, but in this screen, the most striking effect was the near-absolute dependence of MIR2-mediated CD86 downregulation on D244 in the extracellular JM region. This finding agrees with an earlier report that D244 was crucial for MIR2 recognition of a chimeric model substrate containing the extracellular and TMDs of CD8 with the JM region and cytoplasmic tail of CD86 (18).

Given the unimpaired MIR2 activity we observed against CD86 PolyVal TMD, we were surprised to find that many different substitutions scattered throughout the TMD rendered CD86 resistant to MIR2. The positions that harbored such substitutions did not cluster on a common helical face and did not show the pattern we observed for MARCH1 at P254, where none of the energetically most favorable TM amino acids (*boxed region*) (24) were as susceptible as P254. The substitutions that conferred resistance to MIR2 and MARCH1 were, in fact, almost completely mutually exclusive. This pattern suggested to us that while no single position in the CD86 TMD formed part of a required recognition motif for MIR2, it was possible to block MIR2 activity through many different types of substitutions (see more on this in the Discussion section).

### The mutual exclusivity of MARCH1- and MIR2-resistant CD86 variants is recapitulated in traditional site-directed mutagenesis experiments

**Figure 6:**
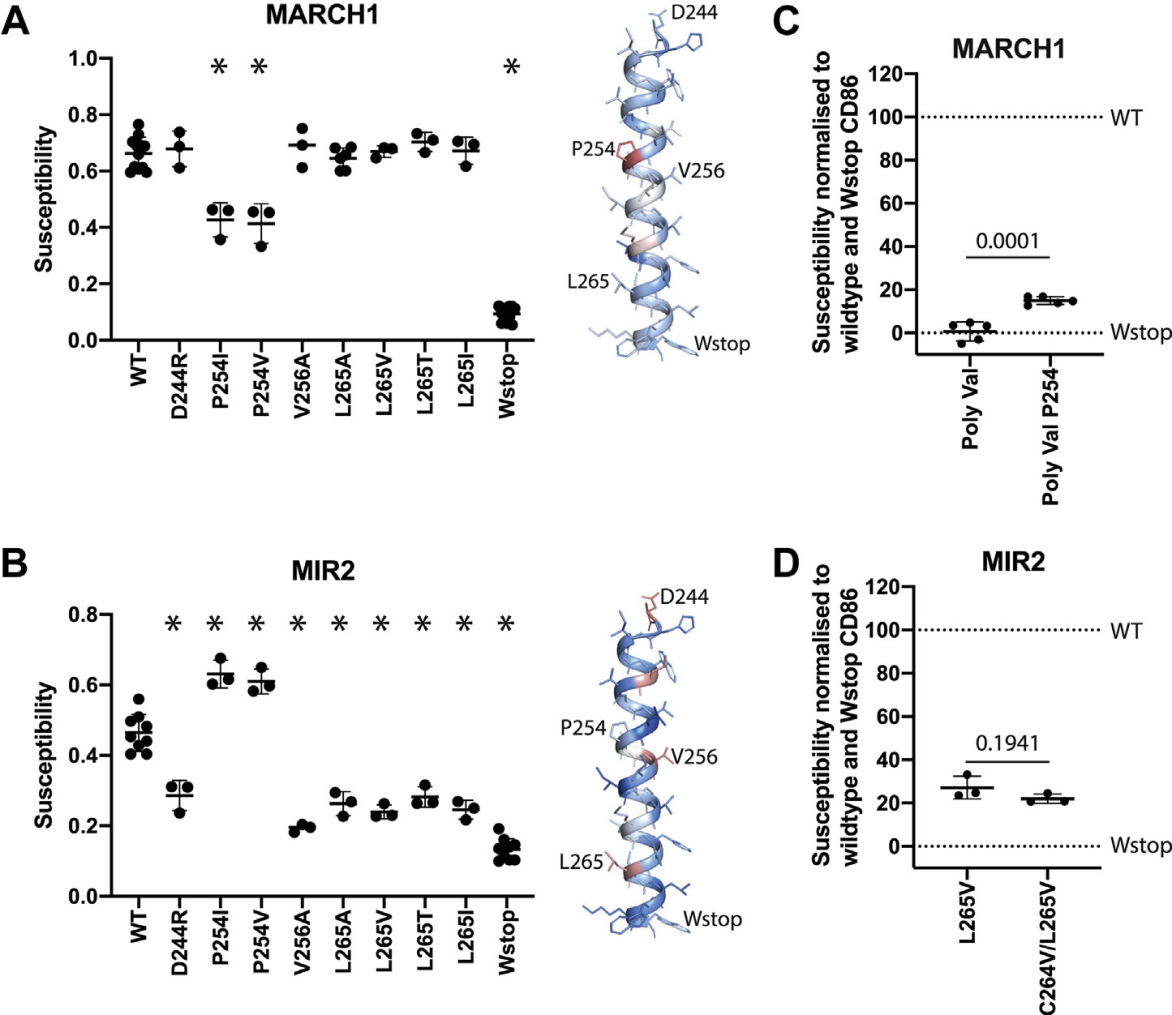
**Mutations selected form MARCH1 and MIR2 sequence-function maps were chosen on the basis that they mitigated downregulation by one E3-ligase but not the other.***A*, in agreement with the data for Figure 4, mutations at position 254 impaired MARCH1-mediated downregulation, but mutations at position 244, 256, and 265 did not. *B*, in agreement with the data in Figure 5, mutations at positions 244, 256, and 265 impaired MIR2-mediated downregulation, but mutations at position 254 did not. In both *A* and *B*, helices from Figures 3 and 4 are duplicated with the positions selected for mutation marked to orient the reader. *C*, installation of a proline at position 254 in the polyvaline CD86 variant restores some susceptibility to MARCH1. *D*, the CD86 C264V/L265V double mutation does not restore susceptibility to MIR2-mediated downregulation and argues against C264 acylation causing altered CD86 trafficking as a mechanism of CD86 L265V evasion of MIR2. *C* and *D* were normalized to downregulation of WT CD86 (100%) *versus* Wstop CD86 (0%), which truncates the cytoplasmic tail of CD86 such that it no longer accepts ubiquitin. Each data point in all graphs represents the mean of technical triplicates in an independent experiment. In *A* and *B*, data that are statistically different from WT are marked with an *asterisk* and were calculated by mixed-effects model with Dunnet correction in Prism with all statistically significant *p* values being less than 0.05. In *C* and *D*, *p* values from unpaired two-tailed *t* tests are reported. MARCH1, membrane-associated RING-CH1; MIR2, modulator of immune recognition 2.

To confirm the key results from the DMS screens, we regenerated a set of resistant variants selected from each screen using traditional site-directed mutagenesis and tested them individually against MARCH1 and MIR2 (Fig. 6, *A* and *B*). The two CD86 variants that conferred the strongest resistance to MARCH1 in the DMS screen, P254(I/V), indeed showed significantly reduced downregulation in the individual tests against MARCH1 (Fig. 6*A*). These had no detrimental effect on MIR2-mediated downregulation (Fig. 6*B*) and may even have increased susceptibility to MIR2. Similarly, substitutions at D244(R), V256(A), and L265(A/V/T/I) selected from the MIR2 DMS screen showed strong reductions in susceptibility to MIR2 in individual tests (Fig. 6*B*) but had no measurable effect on susceptibility to MARCH1 (Fig. 6*A*). The DMS data thus represent an accurate depiction of sequence specificity in downregulation of CD86 by MARCH1 and MIR2 E3 ubiquitin ligases and show that the features they recognize are both starkly contrasting and mutually exclusive.

### P254 alone confers some susceptibility to MARCH1 on the resistant CD86 PolyVal TMD background

The very specific MARCH1 requirement for proline at position 254 in the CD86 TMD prompted us to ask whether P254 was sufficient to restore MARCH1 susceptibility on the background of the otherwise-featureless PolyVal TMD. To test this, we reintroduced a proline residue at position 254 of the PolyVal TMD CD86 construct (PolyVal TMD + P254). In Figure 6*C*, where the downregulation of WT CD86 is set to 100% and the equivalent PolyVal TMD and W270 early termination (Wstop) constructs are set to 0%, the PolyVal + P254 CD86 protein regained a very consistent and statistically significant 15% of the level of downregulation seen for the WT protein. Thus, while P254 alone is not sufficient for full susceptibility to MARCH1, the partial restoration of activity against this protein shows that it makes a significant contribution to a specific molecular surface that is recognized by MARCH1.

### Inhibition of MIR2 activity by L265 mutations is not because of acylation at C264

The apparent blockade of MIR2 activity by substitutions in the CD86 TMD could arise from several possible mechanisms (see Discussion section). In considering the TM site that harbored the highest number of resistant substitutions (L265, with eight different amino acids that were reduced in MIR2-mediated downregulation), we noted that it is directly adjacent to a cysteine residue (C264) close to the cytoplasmic side of the CD86 TM helix. Such cysteine residues are frequently found in membrane proteins and are often sites for S-acylation (most commonly palmitoylation), which can regulate membrane protein trafficking (26). Mutations at C264 did not interfere with MIR2-dependent downregulation (there is no enrichment of any C264 variant in the MIR2 DMS screen, Fig. 5), suggesting that acylation at this residue was not required for MIR2 activity. We therefore hypothesized that substitutions at L265 could generate a new acylation site that either altered CD86 trafficking or blocked MIR2 engagement. Acylation motifs are difficult to predict, so we tested this hypothesis empirically by generating a C264V substitution on top of the MIR2-resistant L265V substitution and checking for restoration of susceptibility. The results in Figure 6*D* show no such restoration of susceptibility to MIR2 and indeed no statistically significant difference from L265V alone. We therefore conclude that the mechanism responsible for decreased MIR2 susceptibility of L265 mutants does not involve acylation at C264.

### Levels of total cellular CD86 variants show defects in degradation that mirror their resistance to internalization

**Figure 7:**
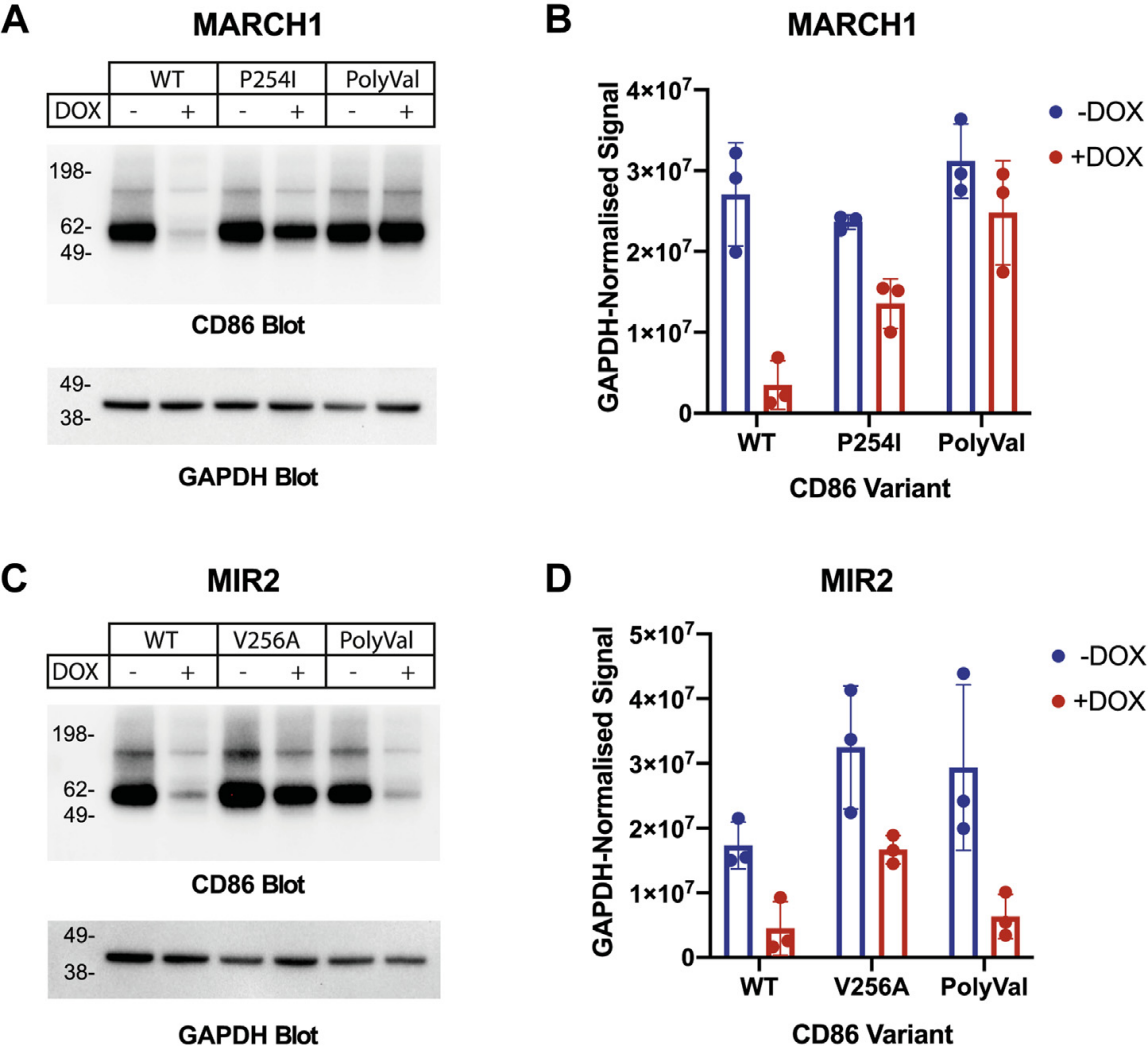
**Levels of total cellular CD86 measured by Western blot for select variants.***A*, CD86 and GAPDH levels were measured by western blot from HeLa whole cell lysates expressing the indicated CD86 variants in the absence (−DOX) or presence (+DOX) of MARCH1. Three separate experimental blots from (*A*) were quantitated by densitometry, and GAPDH-normalized CD86 signals (mean ± standard deviation) from three separate experiments are shown (*B* and *D*). DOX, doxycycline; MARCH1, membrane-associated RING-CH 1; MIR2, modulator of immune recognition 2.

The ultimate consequence of MARCH/MIR-mediated CD86 ubiquitination and internalization is lysosomal degradation (15, 16). To test whether steps downstream of CD86 internalization were also impeded for MARCH1/MIR2-resistant mutants identified by flow cytometry, we selected the mutant with the strongest resistance on each background and examined total cellular CD86 protein levels by Western blot (Fig. 7). HeLa cell lines homogenously expressing P254I with DOX-inducible MARCH1 (Fig. 7, *A* and *B*) or V256A with DOX-inducible MIR2 (Fig. 7, *C* and *D*) were generated by cell sorting, with WT and PolyVal TMD CD86 included as controls on both backgrounds, and whole cell lysates were probed for CD86 before and after Dox treatment. WT and PolyVal TMD CD86 total protein levels perfectly reflect the levels of surface downregulation measured by flow cytometry in Figure 1, *B* and *C*, and each single amino acid mutation exhibited a resistance to degradation that was concomitant with its resistance to surface downregulation measured in the pooled DMS screens (Figs. 4 and 5) and as single mutants assayed independently (Fig. 6). These results are most consistent with a scenario in which substrate recognition is impaired, but all downstream steps including degradation are intact for CD86 molecules that are effectively engaged by MARCH1 or MIR2.

## Discussion

Mammalian MARCH and viral MIR proteins target cell-surface proteins with important immunological functions in order to regulate (MARCH) or evade (MIR) immune responses. The abilities of MARCH1 and MIR2 to internalize and degrade CD86 are strictly dependent on functional E3 ligase domains and lysosomal acidification, demonstrating that they act through a sequence of events involving substrate recognition, ubiquitination on their cytoplasmic tails, internalization, and subsequent lysosomal degradation (11, 15, 16). How these ligases achieve *selectivity* for their membrane protein substrates is less well understood. Early studies showed that substrate specificity could be transferred between the KSHV MIR1 and MIR2 proteins by exchanging their TM–loop–TM regions but not their N- or C-terminal cytoplasmic domains (24). We recently demonstrated that a single serine residue in the first TMD of human MARCH9 (S198) was absolutely required for its activity against multiple substrates, and susceptibility to MARCH9 could be transferred through strict exchange of the TMDs between substrates and nonsubstrates (20). These and similar studies (4, 12, 13, 15, 16, 17, 18, 27) implicate interactions between the membrane-associated TM/JM regions of MARCH-family proteins and their substrates in determining specificity. These observations notwithstanding, there has been little investigation into what *specific* sequences within these regions mark a membrane protein as a substrate. Individual MARCH/MIR proteins can each downregulate a range of targets (28, 29) that seem to have little in common beyond the availability of lysine residues in their cytoplasmic tails and the fact that most are type I single-spanning membrane proteins, raising the question of whether they contain any specific “recognition motifs” at all.

With respect to substrates of mammalian MARCH proteins, only HLA-DRβ has been systematically examined using alanine scanning through the TM/JM regions (17). This study identified the aromatic-rich sequence ^217^LFIYF^221^ at the cytoplasmic TM/JM junction and ^197^SK^198^ in the extracellular JM region as the strongest contributors to MARCH8 susceptibility, suggesting that interactions at both ends of the TMD (but possibly not within the hydrophobic core) were driving interactions with MARCH8. A second study in the same year (18) identified sites in the extracellular JM region of CD86 and the extracellular loop of KSHV MIR2 that made stronger contributions to the CD86–MIR2 interaction than anything in the CD86 TMD, suggesting that regions lying just outside the lipid bilayer can play important, and even dominant, roles in recognition.

Our PolyVal experiment examined strictly TMD contributions to MARCH1 and MIR2 recognition of CD86 and clearly demonstrated that MARCH1 is absolutely dependent on sequences within this region, whereas MIR2 is not. Although a direct structural determination of where the CD86 TMD starts and stops is not available, we considered the predominance of hydrophobic residues between tryptophans at positions 248 and 268, which are common (30) and energetically favorable (24) at the ends of TMDs, to be strong boundary markers indicating which residues are likely to be membrane embedded. CD86 PolyVal TMD protein was expressed at the surface of HeLa cells to normal levels, which in combination with its complete susceptibility to MIR2, rules out a gross defect in stability, membrane insertion, or trafficking.

The DMS screen revealed that human MARCH1 targets a specific recognition sequence in the CD86 TMD whose most important feature is P254, which by itself conferred significant MARCH1 susceptibility on the PolyVal TM background. The pattern of substitutions that conferred resistance, with enrichment scores highest on the same helical face as P254, supports the existence of a contact surface in the core hydrophobic region of the CD86 TMD. At least two possibilities exist with regard to what, exactly, is being recognized at P254. The key unique property of proline in TM helices is that it induces a kink in the α-helix (31, 32) and exposes the backbone carbonyl oxygen at the *i-4* position (T250 in CD86) because proline has no amide proton to serve as a hydrogen-bond donor. This exposed carbonyl oxygen could provide a polar contact site. The small and cyclized side chain can also provide a hydrophobic surface for close van der Waals packing, as observed in the TM trimer structure of the death receptor Fas (CD95, TNFRSF6) (33). No common TM amino acids (*boxed region*) at position 254 allowed WT levels of downregulation by MARCH1, consistent with a unique requirement for some feature of proline. Those substitutions that were tolerated at this site (H/K/R/E/N/Q/W) invariably contain polar moieties in their side chains, suggesting that an electrostatic or hydrogen-bonding interaction may be sufficient in the absence of proline, and this implicates the exposed backbone carbonyl oxygen at T250 in the WT CD86 sequence. Interestingly, human Fas, which is also downregulated by MARCH1 in HeLa cells (3), has a similarly placed proline in the +7 position from tryptophan at the extracellular TM boundary (this distance is +6 in human CD86), but proline is not a feature of all reported MARCH1 substrate TMDs. MARCH1 targets MHC-II for downregulation and, though rich in glycines, these TMDs do not contain any pralines, and MARCH1 recognition has been localized to the C terminus of the HLA-DRβ chain (17).

The MIR2 DMS results confirmed the central importance of D244 in the extracellular JM region of CD86 that was reported previously in a study of chimeric CD8α/CD86 substrates (18). Interestingly, this study reported that the CD86 TMD also contributes to recognition by MIR2: transfer of the CD86 cytoplasmic tail to CD8α (which is not a MIR2 substrate) was not sufficient to confer susceptibility to MIR2, but the CD86 TM and cytoplasmic tail conferred approximately 40% of WT CD86 downregulation. Our PolyVal TM experiment clearly demonstrates that there is no specific contribution from this region to MIR2 recognition, and the surprising DMS result that many CD86 TM substitutions could block downregulation may provide an explanation for this discrepancy. If D244 is the key recognition site and the CD86 TMD need only be *permissive* (as opposed to *required*) for MIR2 action, then a similarly placed CD8α aspartic acid that ends up in the −6 position relative to W248 in the chimeric substrate (as compared with −4 in WT CD86) could be sufficient for 40% downregulation when the permissive CD86 TMD is grafted in. The WT CD8α TM sequence, like many of our MIR2-resistant CD86 TM variants in the DMS screen, must therefore contain features that actively block this recognition and thereby confer resistance.

How, then, do single amino acid TM substitutions in full-length CD86 block downregulation by MIR2? The pattern of resistant variants in the MIR2 DMS screen is very different from the pattern observed in the MARCH1 screen. Sites where substitutions confer resistance are not concentrated in the central hydrophobic region of the CD86 TMD and are not localized to a single helical face. Furthermore, no single TM position bears the signature of a highly specific recognition site like P254 in the MARCH1 screen, where none of the most common TM residues could substitute, and there are many more substitutions that replace hydrophobic or weakly polar residues with strongly polar or ionizable residues. We propose that many of these substitutions act by altering the CD86 TM helix tilt or register in the membrane (by interfering with the match between the length of the hydrophobic core of CD86 and the width of the lipid bilayer (34)) or by imposing new homomeric or heteromeric TM interactions (35, 36, 37) that block access to the key recognition site at D244. Alterations in trafficking are also possible mechanisms of MIR2 evasion, and we explored the possibility that a new acylation site was generated by substitutions at L265 adjacent to C264. While this hypothesis was not borne out by the results of a double C264V/L265V mutation in CD86, MIR2 itself is palmitoylated (38), and mutation of its palmitoylation site C146 blocks downregulation of some substrates because of MIR2 mislocalization. It is therefore possible that any TM substitution that alters CD86 trafficking and/or partitioning to particular membrane subcompartments could result in failure to encounter MIR2 and thereby confer resistance. MIR2 and MARCH1 may have different sensitivity to this type of mutation because of their distinct subcellular localization: MARCH1 is primarily endosomal/lysosomal at steady state, whereas MIR2 is found mostly in the endoplasmic reticulum (1, 11, 39).

It is not known whether substrate recognition by MARCH/MIR proteins relies on direct TM/JM interactions or whether they act through an as-yet unidentified intermediary, and while direct interactions would provide the simplest explanation, our experimental design and analysis neither presume nor exclude either possibility. We do provide strong evidence that substrate recognition, whether direct or indirect, is the step where defects are introduced by TM alterations. The Western blot analysis in Figure 7 effectively rules out a scenario where, for example, substrate binding and internalization are perfectly intact but CD86 variants cannot be degraded and are partially recycled to the membrane, which could give rise to a reduction in cell-surface levels but unperturbed total protein levels. The very strong concordance between surface CD86 levels and total protein levels for the variants we analyzed indicates that internalized proteins do go on to be efficiently degraded. While we have not directly examined ubiquitination or sensitivity to lysosomal inhibitors here, previous studies have firmly established the dependence of internalization and degradation on E3 ligase activity and lysosomal acidification (11, 15, 16). As discussed previously, an important limitation of our study is that we cannot definitively distinguish between failure to bind substrate and failure to encounter substrate at all, a distinction that will require comprehensive analysis of trafficking and intracellular localization for both ligase and substrate variants in future studies.

Our results highlight the evolutionary diversity found within the MARCH/MIR E3 ligases, where functionally homologous mammalian and viral proteins can utilize very different recognition modes to identify and downregulate the same substrate. The combination of reductive (PolyVal TM replacement) and exhaustive (saturating mutagenesis) approaches applied here yields a detailed and multidimensional view of sequence dependence in MARCH/MIR substrate identification that demonstrates how individual mutations and domain swap experiments must be analyzed very carefully when scanning for recognition motifs. In particular, the MIR2 results show that diminished activity against any single variant could represent either loss of a key contact within a crucial binding surface or introduction of a new feature that confers resistance through any number of possible alternative mechanisms. We expect that a similar approach applied to multiple substrates for each MARCH/MIR protein would yield a comprehensive view of molecular recognition modes within the family, opening the possibility of predicting additional natural substrates or designing synthetic binders that could modulate the activities of individual family members with high specificity for research or therapeutic purposes.

## Experimental procedures

### Plasmids and cell lines

Full-length human CD86 was expressed in the lentiviral pHAGE-eF1α-MCS-IRES-ZsGreen backbone (40). Human MARCH1 and Kaposi's sarcoma virus MIR2 were expressed in the DOX-inducible pFUV1-TetOn-MCS-hUb-rtTAV-P2A-mCherry lentiviral vector (41) (a kind gift from Associate Professor Marco Herold). Lentivirus was produced in human embryonic kidney 293T cells, and activity assays were performed in HeLa cells. HeLa cells were established from Henrietta Lacks without her knowledge or consent in 1951 and have made significant contributions to scientific progress and advances in human health. We are grateful to Henrietta Lacks, now deceased, and to her surviving family members for their contributions to biomedical research. WT human CD86 and PolyVal CD86, MARCH1, and MIR2 coding DNA was ordered from IDT as gblocks for cloning into respective lentiviral vectors. Individual mutations were introduced using overlap PCR.

### Lentivirus production

Human embryonic kidney 293T cells were transfected using calcium phosphate with lentiviral expression and packaging vectors. For pFUV1, lentivirus was packaged with pMDLg/pRRE and pRSV-Rev (42), and for pHAGE, lentivirus was packaged with dR8.91. Both were pseudotyped with VSVg for HeLa cell transduction. Virus was harvested 48 h post-transfection, and polybrene (Sigma) was added to 4 μg/ml. Virus was added to HeLa cells in 6-well plates at 50 to 70% confluence and centrifuged at 2500 rpm for 45 min at 32 °C (Heraeus Multifuge 3SR Plus). Transduction efficiency was ascertained by measuring ZsGreen and mCherry expression by flow cytometry.

### Assay measuring CD86 susceptibility to MARCH1 and MIR2

To determine CD86 susceptibility to MARCH1 and MIR2, transduced HeLa cells were treated with 500 ng/ml DOX (Sigma) for 2 days and stained for surface CD86 using Alexa Fluor 647–conjugated mAb IT2.2 (Biolegend). Cells were gated as in Fig. S1 and comprise ZsGreen^+^/mCherry^−^ (CD86^+^ cells that do not express E3 ligase, Ligase^−^), ZsGreen^+^/mCherry^+^ (CD86^+^ cells that do express E3 ligase, Ligase^+^), and ZsGreen^−^/mCherry^−^ (to correct for cellular autofluorescence, ZsGreen^−^). The geometric mean (mean fluorescence intensity [MFI]) of CD86 staining for each of these populations was combined in the following equation to describe CD86 susceptibility.

$$\text{Susceptibility}=1-\left(\left(\text{Log}\left({\text{MFI:Ligase}}^{+}\right)-\text{Log}\left({\text{MFI:ZsGreen}}^{-}\right)\right)/\left(\text{Log}\left({\text{MFI:Ligase}}^{-}\right)-\text{Log}\left({\text{MFI:ZsGreen}}^{-}\right)\right)\right)$$

A susceptibility score of 1 indicates that all CD86 is depleted from the cell surface by MARCH1/MIR2, whereas a score of 0 indicates that CD86 is completely resistant to downregulation.

### CD86 library construction

Each TMD mutation was introduced one by one using primers that had NNN mixed bases at the codon of interest (IDT). An invariant forward primer was mixed with the mutagenesis reverse primer in a PCR to make the 5′ fragment encoding the extracellular and partial TMD. A second PCR with invariant forward and reverse primers was performed in parallel to generate the 3′ fragment encoding the partial TM and full cytoplasmic domain, which overlapped with the 5′ fragment (3′ to the mutated codon). Each PCR product was purified on an agarose gel and used as a template in a third PCR step using invariant forward and reverse primers that flanked the whole insert and installed restriction sites for cloning. The digested PCR product was cloned into pHAGE-eF1a-MCS-IRES-ZsGreen using T4 ligase (New England Biolabs). Ligation reactions were transformed into XL1-Blue *Escherichia coli*, and colonies counted to ensure each position had at least 200 transformants to provide good coverage of 64 possible DNA variants. Colonies were washed directly from LB agar plates into LB broth and grown until cultures were turbid (∼2 h) before plasmid DNA was recovered by miniprep (Qiagen). About 1 μg of DNA from each position was combined to provide a DMS library with complete coverage along the CD86 TMD. As each mutation is cloned individually, our library is devoid of double mutants.

### Library selection

Lentivirus produced from the library was titrated to result in 10% transduction efficiency of 300,000 HeLa cells resulting in 30 to 50,000 transduced cells in each replicate. About 2 days post-transduction, ZsGreen^+^ HeLa cells were sorted from each replicate. These cells were expanded to fill one 6-well plate (∼1 million cells) before mRNA was harvested from half of the cells (Population I), with the remainder sorted for CD86 surface expression. CD86^+^ cells were again grown to confluence before mRNA was harvested from one-third of the cells (population II), with the remainder split in two and transduced with either MARCH1 or MIR2, transducing around half of the cells as measured by mCherry expression. Cells were expanded to confluence and sorted on mCherry expression. This population was expanded, and mRNA was harvested from half of the cells (population III). The other half was incubated with 150 ng/ml of DOX for 48 h to induce MARCH1/MIR2 expression. Cells were stained for CD86 surface expression, and cells containing CD86 variants that were resistant to MARCH1 or MIR2 were recovered and expanded before mRNA was harvested (population IV).

### Amplicon preparation and bioinformatics analysis

About 1 μg of mRNA from 10^6^ cells was reverse transcribed into complementary DNA using a CD86-specific reverse transcription primer (5′-CTGAGACTTG CACATCGCAG C Nx16 ATAAGAGTTG CGAGGCCG-3′) that bound 3′ to the sequence encoding the TMD and contained a 16-bp unique molecular identifier (UMI) and an Illumina adapter for subsequent amplification. The complementary DNA was amplified with the forward primer 5′-GTGACCTATG AACTCAGGAG TCGAGCTTGA GGACCCT-3′ and Illumina reverse primer 5′-CTGAGACTTG CACATCGCAG C-3′ generating a 197 bp product that was multiplexed with Illumina indexing primers in triplicate. A similar amplicon was made directly from the plasmid library using the same forward primer and a reverse primer in which the UMI was absent.

Three unique indexes were added to amplicons derived from each population and replicates for downstream demultiplexing and internal quality control. Samples were paired-end sequenced using an Illumina NextSeq kit with 140 cycles in the forward direction and 160 cycles in the reverse direction. Around 10^6^ overlapping reads per sample were obtained that spanned the region of interest. The paired-end reads from Illumina sequencing runs were separated into samples based on Illumina index sequences using Cutadapt, version 1.15 (43), and forward and reverse reads were merged with USEARCH 9.2.64 (44). Deduplication based on the UMI was performed using UMI Tools, version 1.15 (45), after sample separation. Reads were trimmed to the region of interest and filtered for length using Cutadapt prior to analysis with Enrich2, version 1.2.0 (22).

Log ratio enrichment scores (with WT normalization (22)) were calculated for each of the three replicates to determine expression of CD86 at the cell surface, comparing enrichment of variants in population II over population I, and resistance to MARCH1- or MIR2-mediated downregulation, comparing enrichment of variants in population IV over population III.

### Western blot analysis

HeLa cell lines generated for experiments in Figures 1 and 6 were flow sorted to generate populations of pure CD86+ MARCH/MIR+ cells, and replicate cell samples were cultured in 6-well tissue culture plates with or without 500 ng/ml DOX (Sigma) for 2 days. One well for each condition was lysed in 0.5 ml radioimmunoprecipitation assay buffer containing complete protease inhibitor (P8340; Sigma) for 1 h on ice and then cleared by centrifugation. Samples were mixed with 4× NuPAGE nonreducing lithium dodecyl sulfate sample buffer (Thermo Fisher Scientific), bath sonicated, and heated at 95 °C for 5 min. Lysate samples were separated by electrophoresis in 12% NuPAGE Bis–Tris precast polyacrylamide gels (Thermo Fisher Scientific) at 200 V for 40 min in MES running buffer and transferred to polyvinylidene difluoride membranes that were then blocked in Tris-buffered saline with 0.1% Tween-20 containing 5% w/v bovine serum albumin. CD86 was detected using biotinylated anti-CD86 mAb IT2.2 (Biolegend) and horseradish peroxidase–conjugated streptavidin (Sigma). GAPDH was detected as a loading control using anti-GAPDH-horseradish peroxidase (Sigma). Quantification was performed in Image Lab (Bio-Rad Laboratories), and results were analyzed in Prism 9 (GraphPad Software).

## Data availability

Raw reads are deposited in the Sequence Read Archive (https://www.ncbi.nlm.nih.gov/sra) under accession number PRJNA661137. Processed scores and counts are deposited in the Multiplexed Assays of Variant Effect Database (https://www.mavedb.org) under the accession number urn:mavedb:00000046.

## Supporting information

This article contains supporting information (22).

## Conflict of interest

The authors declare that they have no conflicts of interest with the contents of this article.
