# Supplementary material for: Human and viral membrane–associated E3 ubiquitin ligases MARCH1 and MIR2 recognize different features of CD86 to downregulate surface expression
Source: J Biol Chem. 2021 Jun 19;297(1):100900. doi: 10.1016/j.jbc.2021.100900 (PMC8319528; doi:10.1016/j.jbc.2021.100900)
Supplement: Supplemental Figures S1–S3 [file mmc1.pdf]

Supporting Information

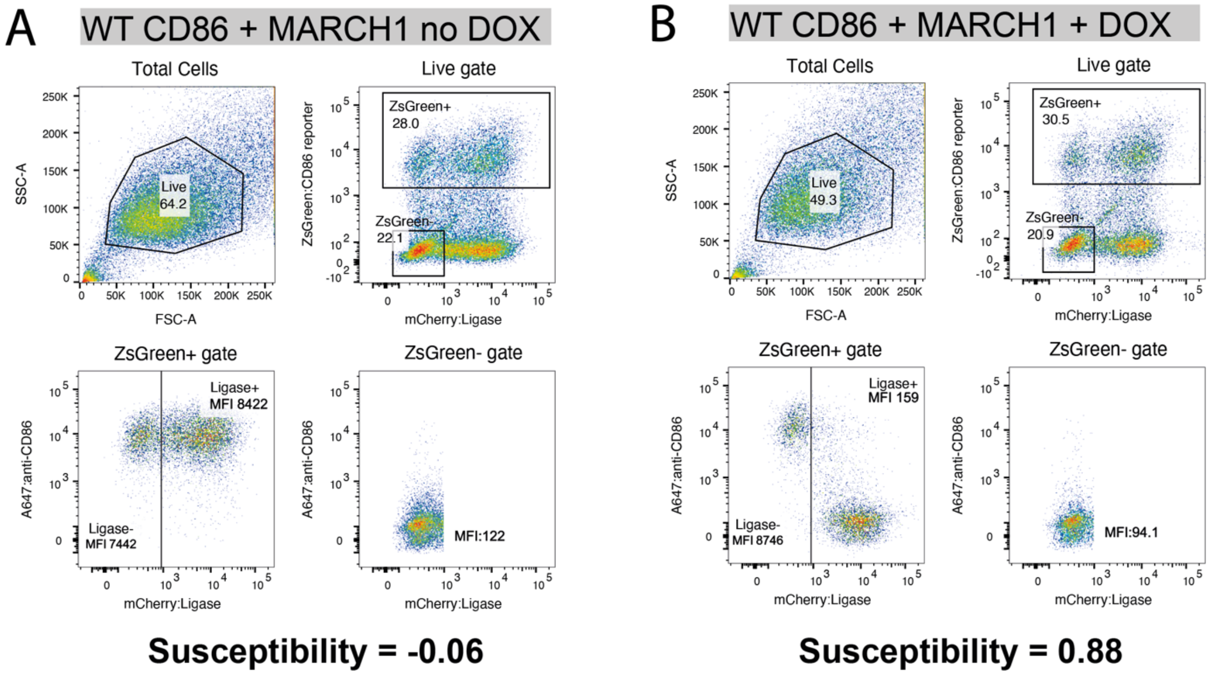

$$\textbf{Susceptibility} = 1 - \left(\frac{\text{Log(MFI:Ligase+) - Log(MFI:ZsGreen-)}}{\text{Log(MFI:Ligase-) - Log(MFI:ZsGreen-)}} \right)$$

Supplementary Figure 1. Gating Strategy and Susceptibility Calculation. Flow cytometry plots showing how susceptibility of CD86 to E3 ligase-mediated downregulation is calculated. (Top) Forward and side scatter was used to gate live cells. (Bottom) Transduction with CD86 and MARCH1/MIR2 was monitored with ZsGreen and mCherry, respectively. Cell surface levels of CD86 were measured by staining cells with Alexa-647 labelled anti-CD86. MARCH1 and MIR2 expression was induced with doxycycline. The ratio of CD86 surface staining of ZsGreen+/mCherry+ cells over ZsGreen+/mCherry- cells, corrected for background CD86 staining in the ZsGreen-/mCherry- population was used to calculate a susceptibility score. **(A)** CD86 is not down-regulated in the absence of doxycycline. **(B)** CD86 is down regulated in MARCH1+ cells when doxycycline is added.

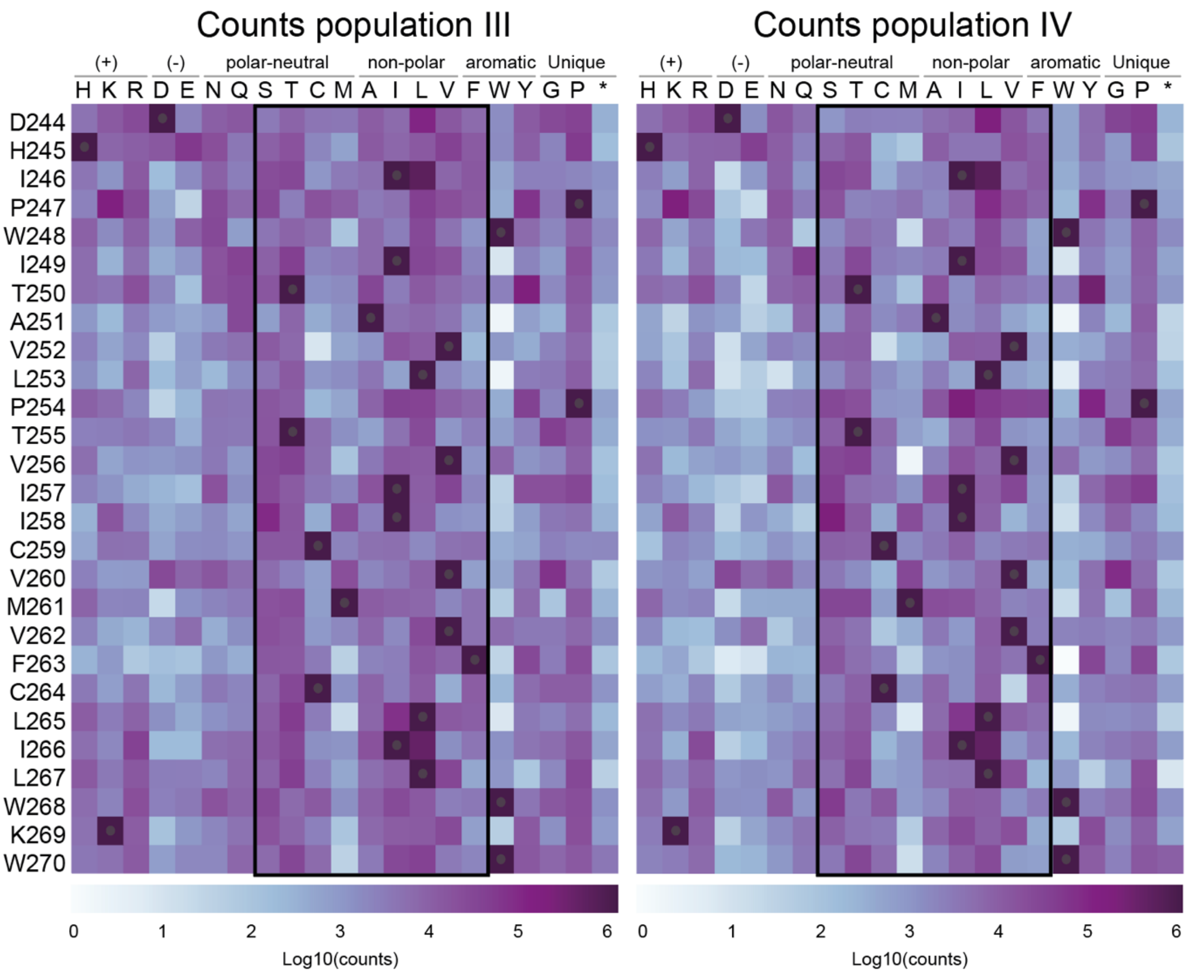

Supplementary Figure 2. Diversity maps of the CD86 deep mutational scanning library before (population III) and after (population IV) MARCH1 induction and flow sorting for cells with CD86 remaining at the cell surface. Maps were calculated in Enrich2 (22) and show the frequency of variants measured by Illumina sequencing in each starting population of cells. The log(counts) scale is shown along the bottom of the heatmap. To generate the sequence-function heat map in Figure 4, each variant was given a log ratio score equal to log(variant counts/wild-type counts). Log ratio scores in the input population were subtracted from the log ratio scores of the selected population to yield the values presented in Figure 4.

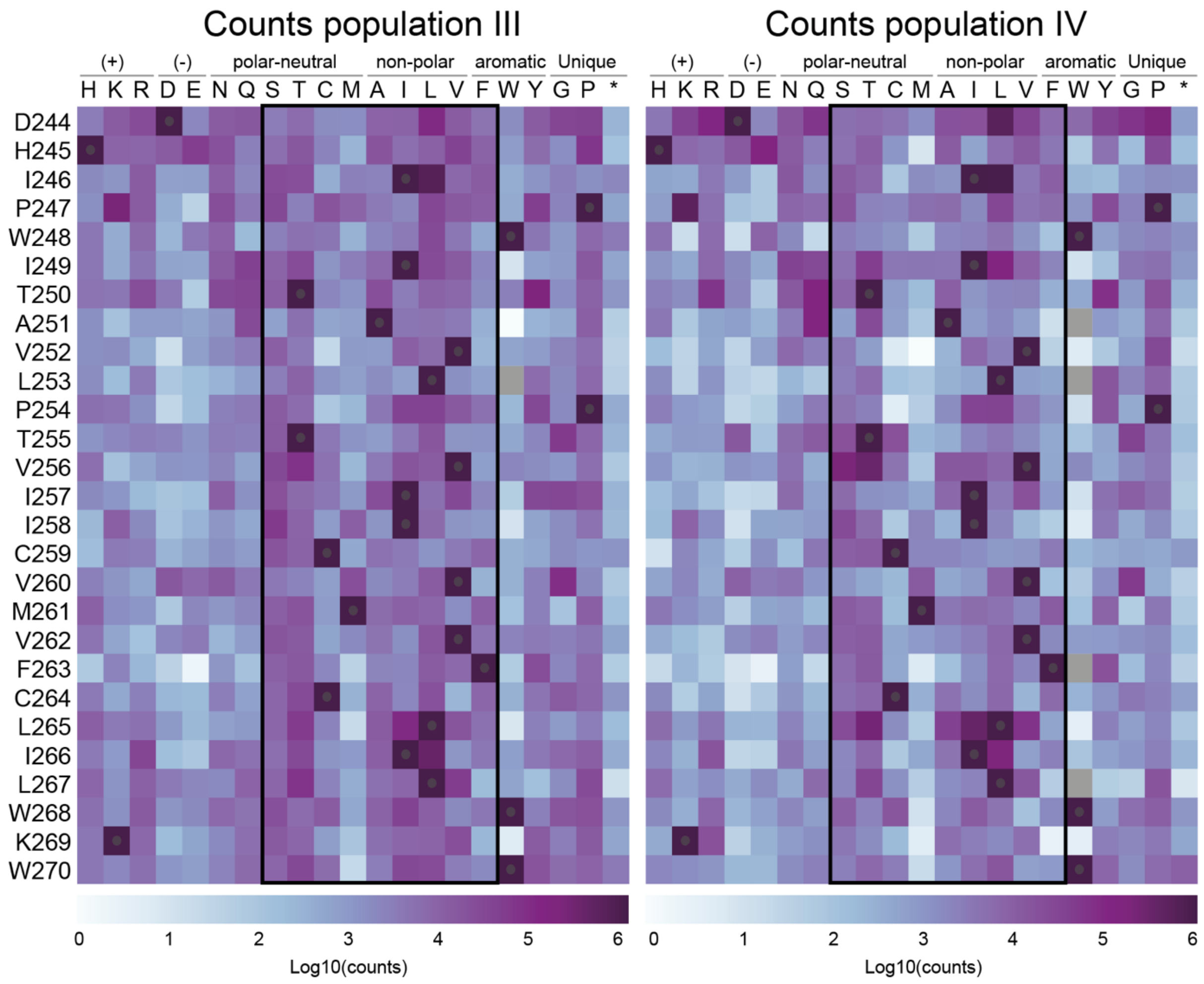

Supplementary Figure 3. Diversity maps of the CD86 deep mutational scanning library before (population III) and after (population IV) MIR2 induction and flow sorting for cells with CD86 remaining at the cell surface. Maps were calculated in Enrich2 (22) and show the frequency of variants measured by Illumina sequencing in each starting population of cells. The log(counts) scale is shown along the bottom of the heatmap. To generate the sequence-function heat map in Figure 5, each variant was given a log ratio score equal to log(variant counts/wild-type counts). Log ratio scores in the input population were subtracted from the log ratio scores of the selected population to yield the values presented in Figure 5.
